# Ingesting carbonated water post‐exercise in the heat transiently ameliorates hypotension and enhances mood state

**DOI:** 10.1113/EP091925

**Published:** 2024-08-14

**Authors:** Masanobu Kajiki, Akira Katagiri, Ryoko Matsutake, Yin‐Feng Lai, Hideki Hashimoto, Takeshi Nishiyasu, Naoto Fujii

**Affiliations:** ^1^ Institute of Health and Sport Sciences University of Tsukuba Tsukuba Japan; ^2^ Research & Development Strategy Department, Research & Development Headquarters Asahi Soft Drinks Co. Ltd Moriya Japan; ^3^ Advanced Research Initiative for Human High Performance (ARIHHP) University of Tsukuba Tsukuba Japan

**Keywords:** cerebral circulation, heat stroke, hydration, pressor response, syncope, thermoregulation

## Abstract

The objective was to assess if post‐exercise ingestion of carbonated water in a hot environment ameliorates hypotension, enhances cerebral blood flow and heat loss responses, and positively modulates perceptions and mood states. Twelve healthy, habitually active young adults (five women) performed 60 min of cycling at 45% peak oxygen uptake in a hot climate (35°C). Subsequently, participants consumed 4°C carbonated or non‐carbonated (control) water (150 and 100 mL for males and females regardless of drink type) at 20 and 40 min into post‐exercise periods. Mean arterial pressure decreased post‐exercise at 20 min only (*P* = 0.032) compared to the pre‐exercise baseline. Both beverages transiently (∼1 min) increased mean arterial pressure and middle cerebral artery mean blood velocity (cerebral blood flow index) regardless of post‐exercise periods (all *P* ≤ 0.015). Notably, carbonated water ingestion led to greater increases in mean arterial pressure (2.3 ± 2.8 mmHg vs. 6.6 ± 4.4 mmHg, *P *< 0.001) and middle cerebral artery mean blood velocity (1.6 ± 2.5 cm/s vs. 3.8 ± 4.1 cm/s, *P* = 0.046) at 20 min post‐exercise period compared to non‐carbonated water ingestion. Both beverages increased mouth exhilaration and reduced sleepiness regardless of post‐exercise periods, but these responses were more pronounced with carbonated water ingestion at 40 min post‐exercise (mouth exhilaration: 3.1 ± 1.4 vs. 4.7 ± 1.7, *P* = 0.001; sleepiness: −0.7 ± 0.91 vs. −1.9 ± 1.6, *P* = 0.014). Heat loss responses and other perceptions were similar between the two conditions throughout (all *P* ≥ 0.054). We show that carbonated water ingestion temporarily ameliorates hypotension and increases the cerebral blood flow index during the early post‐exercise phase in a hot environment, whereas it enhances mouth exhilaration and reduces sleepiness during the late post‐exercise phase.

## Abstract (highlights)

- **What is the central question of this study?**
  Post‐exercise hypotension can cause syncope, and an established practical countermeasure to mitigate this response is currently lacking: does ingestion of carbonated water restore post‐exercise hypotension and positively modulate mood states?
- **What is the main finding and its importance?**
  Carbonated water ingestion transiently increased blood pressure and cerebral blood flow index post‐exercise in the heat. Additionally, carbonated water ingestion increased exhilaration for the mouth and decreased sleepiness post‐exercise. Thus, carbonated water ingestion may mitigate post‐exercise hypotension, increase cerebral perfusion and improve mood states.

## 1 INTRODUCTION

Prolonged exercise in the heat induces post‐exercise hypotension (Cunha et al., 2020; Gagnon et al., 2012; Horiuchi & Oliver, 2023), a response characterized by sustained reductions in arterial blood pressure post‐exercise compared to pre‐exercise resting states. This hypotension could reduce cerebral perfusion, thereby causing reduced orthostatic tolerance or syncope (Cunha et al., 2020; Halliwill et al., 2013, 2014; Horiuchi & Oliver, 2023). One possible countermeasure is drinking water (Halliwill et al., 2014), which may induce a pressor response (Endo et al., 2001, 2002; Fujii et al., 2022; Kubota et al., 2022), possibly through the swallowing reflex (Burke et al., 2020), thereby increasing cerebral blood flow index (Fujii et al., 2022). Interestingly, carbonated water enhances the drink‐mediated pressor responses (Fujii et al., 2022; Kubota et al., 2022), likely due to the augmentation of the swallowing reflex through the activation of TRPV1 channels (Hossain et al., 2018; Tsuji et al., 2020) and/or the activation of nociceptors in the oral cavity (Dessirier et al., 2000). Therefore, carbonated water ingestion may be a more suitable countermeasure against post‐exercise hypotension. One consideration is that the responses mediated by drinking non‐carbonated water or carbonated water mentioned above are transient in healthy young individuals (Endo et al., 2001, 2002; Fujii et al., 2022; Kubota et al., 2022), conceivably due to the augmentation of arterial baroreflex control of muscle sympathetic nerve activity (Vianna et al., 2018). However, since the arterial baroreflex is reset, resulting in less sympathetic outflow and attenuated transduction of sympathetic activity to vascular resistance post‐exercise compared to resting states (Halliwill et al., 1996), drinking water post‐exercise may prolong the pressor response and increase cerebral blood flow index, and these responses may be greater when ingesting carbonated water. These possibilities remain to be investigated.

During prolonged exercise in the heat, the heat loss responses of sweating and cutaneous vasodilatation are activated to increase heat dissipation, thereby avoiding excessive hyperthermia. Upon cessation of exercise in the heat, however, the augmented heat loss responses rapidly subside back to pre‐exercise levels despite sustained hyperthermia (Kenny & McGinn, 2017). Although not always observed (Kenny & Journeay, 2010), increasing arterial pressure by applying positive pressure to the lower limbs, head‐down tilt, or supine recovery augments skin blood flow and sweat rate post‐exercise, possibly through a non‐thermal baroreflex mechanism (Jackson & Kenny, 2003; Kenny et al., 2008; McInnis et al., 2006). Since drinking both carbonated and non‐carbonated water mediates the pressor response with carbonated water exhibiting a greater response as noted above, they may enhance heat loss responses post‐exercise via a non‐thermal baroreflex mechanism, with carbonated water exhibiting greater effects. However, this needs to be directly assessed.

Carbonated water ingestion can modulate several mood states and perceptions (Smit et al., 2004). For example, carbonated water ingestion increases fullness (Wakisaka et al., 2012) but reduces the sensation of thirst (Des Gachons et al., 2016). Additionally, carbonated water ingestion increases exhilaration and motivation while decreasing sleepiness more than non‐carbonated water ingestion under ambient heat exposure at rest (Fujii et al., 2022). Prolonged exercise may also alter moods such as joy, euphoria, cooperation, conscientiousness, and pain sensation to thermal and ischaemic stimulation partially via endogenous opioid neural systems (Janal et al., 1984). Additionally, thermal perceptions and mood state, including thermal sensation and comfort, fatigue and vigor, can be negatively influenced by passive heat stress and acute exercise in the heat (Schlader et al., 2015; Willmott et al., 2020). However, a direct assessment is required to determine whether carbonated water ingestion modulates mood states and perceptions after exercise.

In the current study, we tested the hypothesis that drinking carbonated and non‐carbonated water increases blood pressure, cerebral blood flow index, sweating and cutaneous vasodilatation, and positively modulates thermal perceptions and mood after exercise in the heat. We also investigated whether these responses are greater when carbonated water is consumed. Since exercise‐induced increases in body temperatures, cardiovascular responses, and heat loss responses gradually return to pre‐exercise levels over the course of the recovery period (Cunha et al., 2020; Gagnon et al., 2012; Horiuchi & Oliver, 2023), we assessed the effects of drinking carbonated and non‐carbonated water at 20 and 40 min to evaluate potential time‐dependent modulation. Considering that carbonated water ingestion is easily applicable to various populations without requiring any special devices, in contrast to other interventions for post‐exercise hypotension, such as inspiratory resistance, compression garments and pharmacological approaches (Halliwill et al., 2014), the practical significance of our data seems to be substantial.

## 2 METHODS

### 2.1 Ethics approval

The current study received approval from the Human Subjects Committee of the University of Tsukuba (No. 022‐84) and adhered to the most recent version of the *Declaration of Helsinki* (revised in 2013). Written informed consent was obtained from all participants prior to their participation in the experiments.

### 2.2 Participants

Twelve healthy, habitually active young adults (five women) participated in this study. Based on the differences in elevations in mean arterial pressure elicited by cold carbonated and non‐carbonated water in a previous study (effect size = 1.013) (Kubota et al., 2022), we conducted a power analysis and determined that a minimum of eight participants would be required with at least 80% statistical power (α = 0.05). Participant characteristics, including age, body mass, height and peak oxygen uptake, are presented as means ± standard deviation (SD): 25 ± 5 years, 59.2 ± 6.2 kg, 1.66 ± 0.08 m, and 45.9 ± 11.7 mL/kg/min, respectively. All participants were non‐smokers, and none reported any medical conditions or the use of prescription medications. One of the five females who was using oral contraceptives was tested during the placebo phase. All the other females participated in the experimental session within 10 days of starting menstruation.

### 2.3 Preliminary session

Before the experimental session, all participants underwent an incremental cycling test using a custom semi‐recumbent cycle ergometer (Model 818E, Monark, Stockholm, Sweden) to ascertain their peak oxygen uptake. The test took place in an environmental chamber (Fuji Medical Science Co., Ltd, Chiba, Japan) maintained at 20.0°C and 50.0% relative humidity. Initial workloads were set at 90 W for men and 30 W for women, increasing by 15 W every 1 min. Participants maintained a consistent pedaling rate of 60 rpm until volitional fatigue or until a pedaling rate of <55 rpm was no longer sustainable. Expired volume and gases were continuously analysed using a metabolic cart (AE310s, Minato Medical Science, Osaka, Japan). Peak oxygen uptake was defined as the highest 1‐min oxygen uptake recorded.

### 2.4 Experimental session

The experimental session protocol is depicted in Supporting information, Figure S1. Participants were instructed to refrain from strenuous exercise, alcohol and caffeine consumption for 24 h prior to the session, and to consume the same light meal 2 h before the session. To ensure proper hydration, participants were required to drink 500 mL of water the night before the experiment and an additional 500 mL of water at least 2 h before the session on the day of the experiment. Upon arrival at the laboratory, participants emptied their bladders, and urine samples were collected to assess urine specific gravity (<1.025) (Kenefick & Cheuvront, 2012) using a digital urine specific gravity scale (T3‐SE, ATAGO, Tokyo, Japan). Additionally, participants’ body mass was measured using a scale (model ICS429, Mettler Toledo, Schwerzenbach, Switzerland), and they were instructed to self‐insert a rectal probe (model 401, NikkisoTherm Co., Ltd, Tokyo, Japan). Subsequently, participants entered an environmental chamber (Fuji Medical Science Co., Ltd) set at 35°C ambient temperature and 50% relative humidity, where they rested on the seat of a semi‐recumbent cycle ergometer (Model 818E) for 60 min while attaching the equipment. After instrumentation, pre‐exercise resting data were collected for 20 min. During the pre‐exercise measurements, blood samples were obtained from a warmed fingertip. Participants then engaged in semi‐recumbent cycling exercise at approximately 45% of their individual predetermined peak oxygen uptake, maintaining a pedaling rate of 60 rpm. Five min after the exercise, fingertip blood samples were collected. Participants rested in a semi‐recumbent position for 50 min post‐exercise, during which they consumed 4°C carbonated water (Wilkinson, Asahi Soft Drink Co., Ltd, Tokyo, Japan) using a straw within approximately 30 s at 20 and 40 min into the post‐exercise period (defined as the early and late post‐exercise periods). The temperature of the drinks was maintained at 4°C because cooler beverages are typically consumed in real life. We did not assess post‐exercise responses for more than 50 min, as physiological and perceptual responses exhibited little change beyond this period. At the end of the 50‐min post‐exercise recovery period, finger blood samples were collected, body mass was recorded, and thereafter urine samples were collected. On a separate day, a control trial was conducted in which participants consumed 4°C non‐carbonated water (Oishii Mizu, Asahi Soft Drink Co.) instead of carbonated water. Participants were blinded to the order of the trials, and the two trials were separated by at least 5 days. The order of the two trials was randomized and counterbalanced. The two trials were conducted at the same time of day. The volume of each drink consumed at each post‐exercise time point was 150 mL for men and 100 mL for women, following our previous work (Fujii et al., 2022).

### 2.5 Measurements

#### 2.5.1 Cardiovascular responses

Using the volume clamp method, arterial blood pressure was measured beat‐to‐beat using a finger photoplethysmography system (Finometer, Finapres Medical System, Amsterdam, Netherlands) on the left middle finger, placed at heart level. Mean arterial pressure was estimated as diastolic arterial pressure plus one‐third of the pulse pressure using a software calculation (LabChart, ADInstruments, Bella Vista, NSW, Australia). Finger blood pressures were corrected using arterial blood pressure in the contralateral upper arm measured with an automated sphygmomanometer (Tango M2, Sun Tech Medical, Durham, NC, USA). Middle cerebral artery blood velocity was measured beat‐to‐beat using a transcranial Doppler ultrasound system (EZ‐Dop, Compumedics Germany GmbH, Singen, Germany). A 2‐MHz probe was positioned in the left cranial temporal bone window, with sample volume depth and width set at 45–55 and 8 mm, respectively. Analog data were digitized using an AD converter (PowerLab, ADInstruments) at a sampling frequency of 200 Hz. Cerebrovascular conductance index was evaluated as the middle cerebral artery mean blood velocity divided by mean arterial pressure. Stroke volume was estimated from finger blood pressures using the model flow method (Wesseling et al., 1993). Due to technical difficulties, stroke volume in two participants could not be successfully measured, resulting in the analysis of data from 10 participants. Heart rate was recorded every 5 s using a heart rate monitor (RS400 or VANTAGE V2, Polar, Kempele, Finland) for 10 participants. Equipment failure prevented the acquisition of heart rate data in two participants, where heart rate was instead derived from the arterial waveform recorded by the finger photoplethysmography system mentioned earlier. Cardiac output was calculated as the product of stroke volume and heart rate. Total peripheral resistance was determined by dividing mean arterial pressure by cardiac output.

Skin blood flow on the left dorsal forearm and left chest was assessed using a laser‐Doppler flowmeter (ALF21, Advance, Tokyo, Japan), with values presented in millivolts. A custom‐made integrated laser‐Doppler probe (Ø 4.0 mm, Advance) was employed for this purpose. The probe consists of a central laser irradiation component and four laser‐receiving components surrounding it, housed in a Peltier unit (26.5 mm width, 35 mm dimension, 19.6 mm height, 90 g, ADT1045, Advance). It can evaluate skin blood flow across a circular area with a diameter of 2.0 mm. The Peltier unit with the laser‐Doppler probe was affixed to the skin using double‐sided tape and surgical tape. Cutaneous vascular conductance was calculated as the laser‐Doppler signal divided by mean arterial pressure to account for perfusion pressure differences.

#### 2.5.2 Sweating responses

Sweat rates were determined using the sweat capsule method. A sweat capsule with a 2.83 cm^2^ surface area was affixed to the left forearm and chest. Dry compressed N_2_ gas, equilibrated to 25°C, was supplied from gas tanks to each capsule at a rate of 1.0 L/min, regulated by a mass flow controller (RK‐1200, Kofloc, Kyoto, Japan). A capacitance hygrometer (HMT330, Vaisala, Helsinki, Finland) measured relative humidity and temperature in the effluent air from the sweat capsule every 1 s, recorded in the data logger (GM10, Yokogawa, Tokyo, Japan). Long vinyl tubes ensured connections between the N_2_ gas tank and the sweat capsule (inlet) and between the sweat capsule and the capacitance hygrometer (outlet). The N_2_ flow rate, capsule surface area, and measured relative humidity and temperature were utilized to calculate local sweat rate (mg/cm^2^/min) (Fujii et al., 2019). Due to technical difficulties, the sweat rate on the chest of one participant could not be obtained (*n* = 11).

#### 2.5.3 Body temperatures

Rectal temperature was continuously monitored with a thermistor (401, NikkisoTherm Co.), positioned approximately 15 cm from the external anal sphincter. Data for rectal temperature were recorded at 1‐s intervals using a data logger (N543, NikkisoTherm Co.). Due to unsuccessful measurement in one participant, data from 11 participants were analysed. Skin temperature was assessed using thermocouples affixed to seven skin sites on the left side: the forehead, chest, forearm, hand, thigh, calf and foot. All skin temperature variables were recorded every 1 s using a data logger (GM10, Yokogawa). Mean skin temperature was computed using the equation proposed by Hardy and Dubois (Hardy et al., 1938). In the case of unsuccessful measurement of foot skin temperature in one participant, mean skin temperature was determined using an alternative equation proposed by Ramanathan (1964).

#### 2.5.4 Respiratory responses

The respiratory variables (minute ventilation, oxygen uptake and CO_2_ elimination) were measured using the same analysers employed in the preliminary session. The end‐tidal CO_2_ partial pressure, an index of arterial CO_2_ partial pressure, recorded post‐ingestion was not used in this study because several artifacts associated with the ingestion of carbonated water were detected on the end‐tidal CO_2_ partial pressure. To acquire inspiratory and expiratory gases, a mask was affixed to the participant's face before and during exercise, and a mouthpiece was attached to the participant's mouth while their nose was closed using a nose clip throughout the post‐exercise period, except during drinking. The face mask was utilized before and during exercise to mitigate the stress associated with prolonged nose clip attachment. The mouthpiece and nose clip were detached 30 s before the commencement of each drinking session and reattached 1 min after its conclusion. Consequently, we were unable to collect respiratory data from 30 s before to 1 min after drinking. Owing to technical difficulties, respiratory variables for one participant could not be successfully measured, and thus we analysed data from 11 participants.

#### 2.5.5 Perceptions and mood states

Thermal sensation was assessed using a 40‐point categorical scale (−20: unbearably cold; −16: very cold; −12: cold; −8: cool; −4: slightly cool; 0: neutral; 4: slightly warm; 8: warm; 12: hot, 16: very hot; 20: unbearably hot) (West et al., 2019). Thermal comfort was evaluated using a nine‐point categorical scale (1: comfortable; 3: slightly uncomfortable; 5: uncomfortable; 7: very uncomfortable; 9: unbearably uncomfortable) (West et al., 2019). We employed a visual analog scale, consisting of a 100‐mm horizontal straight line, representing scores from 0 (none) to 100 (most intense experience), to assess three subjective perceptions: thirst, the stimulating feelings of mouth, and abdominal fullness, along with four mood states: exhilaration for the mouth and whole‐body, whole‐body fatigue, and sleepiness. Participants marked all variables within approximately 30 s on all occasions: pre‐exercise baseline, immediately after exercise, and 1 min before, as well as 1, 5 and 10 min after drinking. All participants were fully informed about the meaning of all perception and mood state scales. During the last 5 min of a 60‐min exercise, thermal sensation, thermal comfort, and rate of perceived exertion (Borg 1998) were assessed.

#### 2.5.6 Blood and urine analyses

Haemoglobin concentration was determined using the azide methaemoglobin method (B‐Hemoglobin, Hemocue, Brea, CA, USA), while haematocrit was measured using the microcentrifuge method, each assessed three times, and the mean value was utilized for data analysis. The percentage change in plasma volume was calculated from haemoglobin concentrations and haematocrits, employing the equation established by Dill and Costill (1974). Blood samples, separated into aliquots, were centrifuged at 4°C, and plasma osmolality was determined three times using the freezing point depression method (Fiske 210 Micro‐Sample Osmometer, Advanced Instruments, Norwood, MA, USA) based on a portion of the separated plasma; the average value was employed for data analysis. In one participant, blood samples were not obtained after the post‐exercise recovery period (*n* = 11). Urine specific gravity was assessed with a refractometer (T3‐SE, Atago Co., Ltd, Tokyo, Japan).

### 2.6 Data analyses

Physiological variables (excluding respiratory variables) were averaged over the final 5 min of the pre‐exercise resting period, the last 1 min of each pre‐drinking period (pre‐drink baseline), and the entire 1‐min drinking period. Changes in (Δ) physiological variables during drinking from the pre‐drink baseline were also calculated for each post‐exercise period. Respiratory variables were averaged over a 1‐min post‐drink period instead of during drinking, as the mouthpiece was removed during drinking. Changes in (Δ) respiratory, perceptual and mood state variables at 1‐min post‐drink period from baseline were calculated for each post‐exercise period. The change in body weight during the experiment was evaluated to estimate the whole‐body sweat rate. Physiological variables including respiratory variables were averaged over the last 5 min of a 60‐min exercise.

### 2.7 Statistics

Data were analysed using statistical software (SPSS version 29.0, IBM Corp., Armonk, NY, USA), and figures were generated using R software (version 4.2.2, R Foundation for Statistical Computing, Vienna, Austria). A two‐way repeated measure analysis of variance (ANOVA) was used for physiological (excluding respiratory responses), perceptual and mood state variables, with repeated factors of drink (water and carbonated water) and time (pre‐exercise baseline and pre‐drink baseline during post‐exercise periods). Respiratory responses assessed with a face mask and mouthpiece were analysed separately due to differences in dead space. Respiratory responses with a face mask were analysed using Student's *t*‐test during the pre‐exercise period, whereas respiratory data measured with the mouthpiece were analysed using a two‐way repeated measures ANOVA with repeated factors of drink (as above) and time (pre‐drink baseline during post‐exercise periods). A two‐way repeated measures ANOVA was also employed for the drink‐induced change (Δ) from pre‐drink baseline for physiological, perceptual, and mood state variables, with repeated factors of drink (as above) and time (physiological: pre‐drink baseline and during drinking; perceptual and mood state: pre‐drink baseline and 1 min after drinking). Analyses for Δ values were conducted separately for each of the recovery periods. Blood and urine variables were analysed using a two‐way repeated measures ANOVA with repeated factors of drink (as above) and time (blood: before and immediately after exercise and immediately after the 50‐min post‐exercise recovery; urine: before exercise and after the 50‐min post‐exercise recovery). The normal distribution of variables used for the above analyses was confirmed by two investigators (M.K. and N.F.) using a Q–Q plot, with a few exceptions (e.g., stimulating feelings of the mouth, abdominal fullness and sleepiness). Given that the ANOVA is robust against the normality assumption, we included a few non‐normally distributed data in the ANOVA analyses. The Greenhouse–Geisser correction was applied when the assumption of sphericity was violated in the above ANOVA analyses. All data are presented as means ± SD. A level of *P* < 0.05 was considered statistically significant.

## 3 RESULTS

### 3.1 Baseline data

All physiological, perceptual and mood state variables during all baseline periods did not differ between the non‐carbonated and carbonated water ingestion trials (Tables 1, 2, 3), except for the respiratory exchange ratio at the pre‐exercise baseline (Supporting information, Table S1, *P* = 0.003). Additionally, the sensations of stimulating feelings in the mouth and abdominal fullness at the pre‐second drink baseline were higher in the carbonated water ingestion trial compared to the non‐carbonated one (both *P* < 0.05, Table 3). Pre‐exercise plasma osmolality (Table 4), body mass (58.9 ± 6.6 vs. 59.3 ± 6.5 kg, *P* = 0.121), and urine specific gravity (1.014 ± 0.016 vs. 1.014 ± 0.006, *P* = 0.859) did not differ between the non‐carbonated and carbonated water ingestion trials.

**TABLE 1:** Baseline cardiovascular variables measured immediately before exercise and ingesting first and second drinks.

				ANOVA *P*‐value		
	Pre‐exercise	Pre‐first drink	Pre‐second drink	Trial	Time	Interaction
Mean arterial pressure (mmHg)^†^				0.363	**0.039**	0.756
Water	83.0 ± 10.1	76.5 ± 16.7	79.6 ± 17.5			
Carbonated water	86.1 ± 11.0	78.6 ± 12.7	81.2 ± 15.0			
Middle cerebral artery mean blood velocity (cm/s)				0.299	0.105	0.696
Water	59.9 ± 13.8	55.6 ± 15.4	56.8 ± 15.5			
Carbonated water	57.0 ± 15.0	54.6 ± 11.7	54.6 ± 12.3			
Cardiac output (L/min), *n* = 10				0.805	0.052	0.232
Water	5.1 ± 0.8	6.1 ± 1.8	5.4 ± 1.6			
Carbonated water	5.5 ± 1.3	5.9 ± 1.5	5.5 ± 1.5			
Heart rate (bpm)^†, ‡, ¶^				0.784	**<0.001**	0.379
Water	65.0 ± 9.6	94.9 ± 16.4	86.9 ± 11.9			
Carbonated water	67.1 ± 13.0	96.3 ± 14.0	85.6 ± 11.6			
Stroke volume (mL/beat), *n* = 10^†, ‡^				0.708	**<0.001**	0.094
Water	81.5 ± 14.5	63.8 ± 15.8	61.1 ± 16.1			
Carbonated water	85.3 ± 17.0	61.0 ± 14.1	63.7 ± 14.8			
Total peripheral resistance (mmHg/L/min), *n* = 10^†, ¶^				0.738	**0.011**	0.440
Water	16.5 ± 2.7	13.2 ± 3.1	15.3 ± 3.4			
Carbonated water	16.6 ± 2.5	13.9 ± 2.9	15.1 ± 3.0			

*Note*: Values are presented as means ± SD; *n* = 12 unless otherwise indicated. Values at pre‐exercise, pre‐first drink, and pre‐second drink were obtained by averaging data over the last 5 min of pre‐exercise resting period and 19–20 and 39–40 min into post‐exercise periods. Data were analysed by two‐way repeated measures ANOVA. When an interaction between trial and time was detected, *post hoc* multiple comparisons were performed with Bonferroni correction. ^†^Pre‐exercise versus pre‐first drink regardless of trials (*P* < 0.05). ^‡^Pre‐exercise versus pre‐second drink regardless of trials (*P* < 0.05). ^¶^Pre‐first drink versus pre‐second drink regardless of trials (*P* < 0.05). Bold values indicate statistical significance.

**TABLE 2:** Baseline thermoregulatory and temperature variables measured immediately before exercise and ingesting first and second drinks.

				ANOVA *P*‐value		
	Pre‐exercise	Pre‐first drink	Pre‐second drink	Trial	Time	Interaction
Chest skin blood flow (mV)^†, ‡, ¶^				0.344	**<0.001**	0.127
Water	0.81 ± 0.37	1.84 ± 0.82	1.18 ± 0.64			
Carbonated water	0.78 ± 0.29	2.09 ± 1.01	1.51 ± 0.87			
Chest cutaneous vascular conductance (100*mV/mmHg)^†, ‡, ¶^				0.455	**<0.001**	0.151
Water	0.97 ± 0.44	2.32 ± 0.80	1.43 ± 0.61			
Carbonated water	0.89 ± 0.23	2.58 ± 1.02	1.76 ± 0.76			
Chest sweat rate (mg/cm^2^/min), *n* = 11^†, ‡, ¶^				0.856	**<0.001**	0.995
Water	0.34 ± 0.15	1.29 ± 0.84	0.97 ± 0.67			
Carbonated water	0.32 ± 0.14	1.25 ± 0.56	0.95 ± 0.40			
Forearm skin blood flow (mV)^†, ¶^				0.108	**<0.001**	0.200
Water	0.89 ± 0.35	1.69 ± 0.66	1.28 ± 0.76			
Carbonated water	0.91 ± 0.51	2.04 ± 0.96	1.52 ± 0.86			
Forearm cutaneous vascular conductance (100*mV/mmHg)^†, ‡, ¶^				0.202	**<0.001**	0.150
Water	1.06 ± 0.38	2.17 ± 0.71	1.56 ± 0.80			
Carbonated water	1.04 ± 0.51	2.55 ± 1.12	1.83 ± 0.95			
Forearm sweat rate (mg/cm^2^/min)^†, ‡, ¶^				0.461	**<0.001**	0.510
Water	0.27 ± 0.05	1.09 ± 0.53	0.75 ± 0.40			
Carbonated water	0.28 ± 0.09	1.15 ± 0.56	0.83 ± 0.34			
Rectal temperature (°C), *n* = 11^†, ‡, ¶^				0.554	**<0.001**	0.939
Water	36.89 ± 0.36	38.33 ± 0.45	37.92 ± 0.40			
Carbonated water	36.85 ± 0.28	38.30 ± 0.49	37.87 ± 0.33			
Mean skin temperature (°C)^†, ¶^				0.106	**<0.001**	0.296
Water	35.68 ± 0.44	36.35 ± 0.51	35.69 ± 0.45			
Carbonated water	35.48 ± 0.35	36.23 ± 0.37	35.53 ± 0.32			

*Note*: Values are presented as means ± SD; *n* = 12 unless otherwise indicated. Values at pre‐exercise, pre‐first drink, and pre‐second drink were obtained by averaging data over the last 5 min of pre‐exercise resting period and 19–20 and 39–40 min into post‐exercise periods. Data were analysed by two‐way repeated measures ANOVA. When an interaction between trial and time was detected, *post hoc* multiple comparisons were performed with Bonferroni correction. ^†^Pre‐exercise versus pre‐first drink regardless of trials (*P* < 0.05). ^‡^Pre‐exercise versus pre‐second drink regardless of trials (*P* < 0.05). ^¶^Pre‐first drink versus pre‐second drink regardless of trials (*P* < 0.05). Bold values indicate statistical significance.

**TABLE 3:** Baseline perceptive and mood state variables measured immediately before exercise and ingesting first and second drinks.

				ANOVA *P*‐value		
	Pre‐exercise	Pre‐first drink	Pre‐second drink	Trial	Time	Interaction
Thermal sensation				0.270	0.107	0.103
Water	6.1 ± 2.7	6.0 ± 5.5	2.6 ± 4.6			
Carbonated water	6.3 ± 3.7	6.0 ± 6.3	5.1 ± 4.7			
Thermal comfort^†, ¶^				0.218	**0.001**	0.800
Water	2.6 ± 1.0	3.5 ± 1.4	2.4 ± 1.1			
Carbonated water	2.8 ± 1.6	3.8 ± 1.7	2.8 ± 1.3			
Thirst^†, ‡^				0.725	**<0.001**	0.239
Water	4.1 ± 1.9	6.4 ± 2.6	5.8 ± 2.3			
Carbonated water	3.4 ± 2.4	7.1 ± 1.9	5.4 ± 2.8			
Stimulating feelings of the mouth				0.149	0.380	**0.027**
Water	0.8 ± 1.5	0.5 ± 0.9	0.6 ± 0.8			
Carbonated water	0.5 ± 1.1	0.7 ± 0.9	1.2 ± 1.4*			
Abdominal fullness				0.235	0.220	**0.032**
Water	0.9 ± 1.2	1.4 ± 2.3	0.9 ± 1.3			
Carbonated water	1.2 ± 1.6	1.5 ± 1.9	2.5 ± 2.0*			
Exhilaration for the mouth^¶^				0.113	**0.002**	0.522
Water	0.9 ± 1.1	0.4 ± 0.5	1.7 ± 1.2			
Carbonated water	1.6 ± 1.8	0.7 ± 0.9	1.7 ± 1.3			
Whole body exhilaration^‡^				0.405	**0.015**	0.818
Water	1.0 ± 0.8	1.2 ± 1.3	2.1 ± 2.0			
Carbonated water	1.5 ± 1.5	1.5 ± 1.5	2.0 ± 1.4			
Feeling of fatigue for whole‐body^†, ‡, ¶^				0.157	**<0.001**	0.649
Water	2.0 ± 1.9	4.8 ± 1.9	4.1 ± 2.0			
Carbonated water	2.1 ± 2.1	5.4 ± 1.3	4.9 ± 1.8			
Sleepiness				0.138	0.100	0.091
Water	3.7 ± 2.4	2.6 ± 2.5	3.8 ± 3.2			
Carbonated water	5.5 ± 2.8	2.6 ± 2.3	4.2 ± 2.8			

*Note*: Values are presented as means ± SD; *n* = 12. Values at pre‐exercise, pre‐first drink, and pre‐second drink were obtained at 5 min before the start of exercise and 19 and 39 min into post‐exercise periods. Data were analysed by two‐way repeated measures ANOVA. When an interaction between trial and time was detected, *post hoc* multiple comparisons were performed with Bonferroni correction. ^*^Versus non‐carbonated water at same time point (*P* < 0.05). ^†^Pre‐exercise versus pre‐first drink regardless of trials (*P* < 0.05). ^‡^Pre‐exercise versus pre‐second drink regardless of trials (*P* < 0.05). ^¶^Pre‐first drink versus pre‐second drink regardless of trials (*P* < 0.05). Bold values indicate statistical significance.

**TABLE 4:** Hydration variables.

		Post‐exercise		ANOVA *P*‐value		
	Pre‐exercise	5 min	50 min	Trial	Time	Interaction
Change in plasma volume (%)^†, ‡, ¶^				0.102	**<0.001**	0.320
Water	0 ± 0	−13.4 ± 4.4	−10.5 ± 7.3			
Carbonated water	0 ± 0	−11.4 ± 5.9	−7.3 ± 2.8			
Plasma osmolality (mOsmol/L)^†, ‡^				0.584	**<0.001**	0.218
Water	293.1 ± 4.2	301.3 ± 6.4	296.1 ± 4.4			
Carbonated water	293.4 ± 6.3	300.2 ± 5.2	299.3 ± 6.4			

*Note*: Values are presented as means ± SD; *n* = 11. Values at pre‐exercise and post‐exercise were obtained at 20 min before the start of exercise and 5 and 50 min into post‐exercise periods. Data were analysed by two‐way repeated measures ANOVA. When an interaction between trial and time was detected, *post hoc* multiple comparisons were performed with Bonferroni correction. ^†^Pre‐exercise versus pre‐first drink regardless of trials (*P* < 0.05). ^‡^Pre‐exercise versus pre‐second drink regardless of trials (*P* < 0.05). ^¶^Pre‐first drink versus pre‐second drink regardless of trials (*P* < 0.05). Bold values indicate statistical significance.

### 3.2 Responses during exercise

End‐exercise variables did not differ between the two trials, except for forearm skin blood flow and CO_2_ elimination (both *P* < 0.05, Supporting information, Table S2).

### 3.3 Cardiovascular responses

**FIGURE 1:**
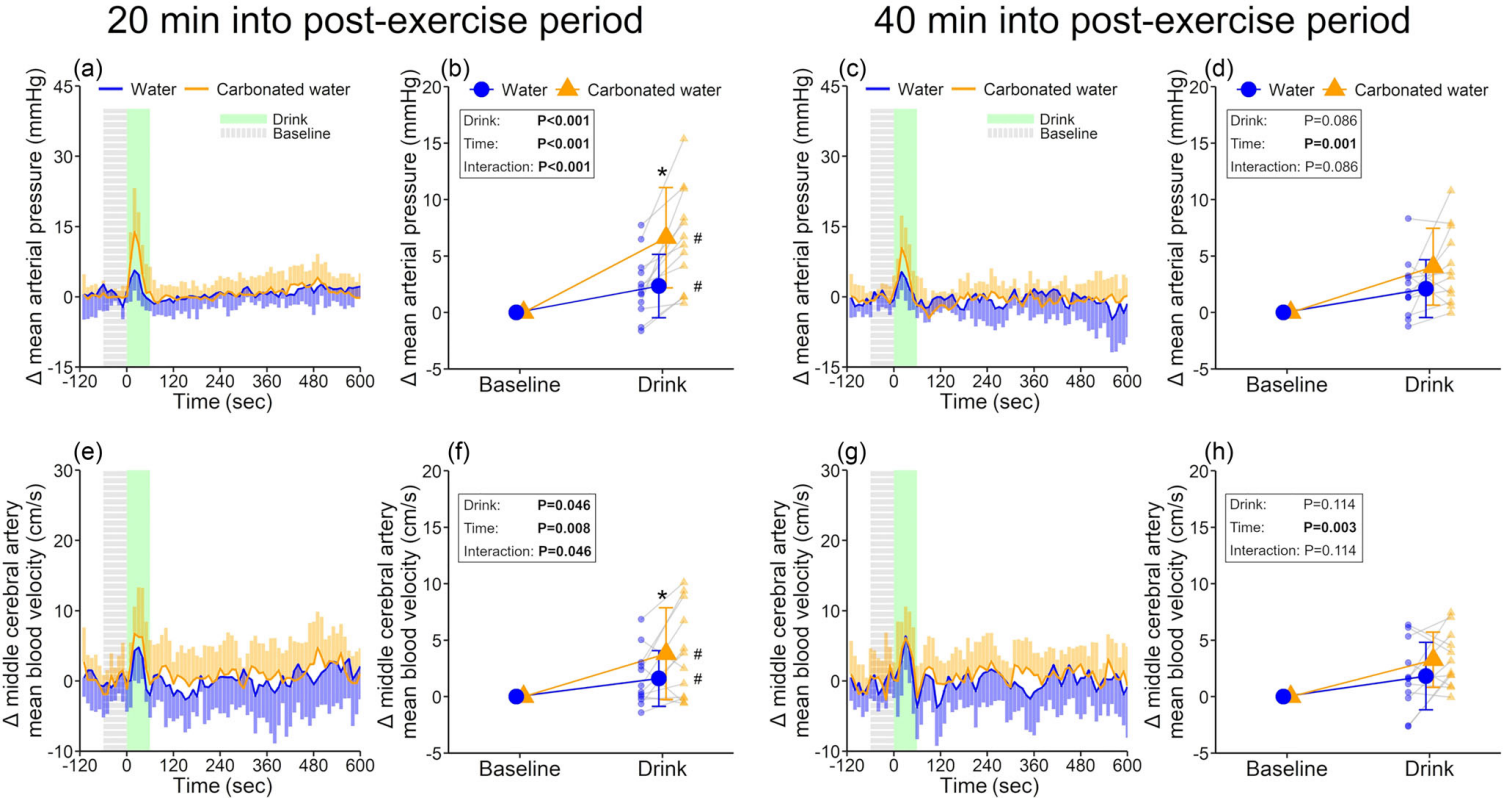
Blood pressure and cerebral blood flow index responses to drinking. Mean arterial pressure (a‐d) and middle cerebral artery mean blood velocity (e‐h) during drink periods (*n* = 12). Every 10‐s data point during drink periods (a, c, e, g) and the drink‐induced change from baseline (b, d, f, h) are presented for both variables. Two‐way repeated measures ANOVA was conducted on the data presented in b, d, f and h. When an interaction between drink and time was detected, *post hoc* multiple comparisons were carried out with Bonferroni correction. *Versus non‐carbonated water (*P* < 0.05); #versus baseline within a trial (*P* < 0.05). Data are presented as means ± standard deviation with individual data points represented in b, d, f and h. Bold values indicate statistical significance.

**FIGURE 2:**
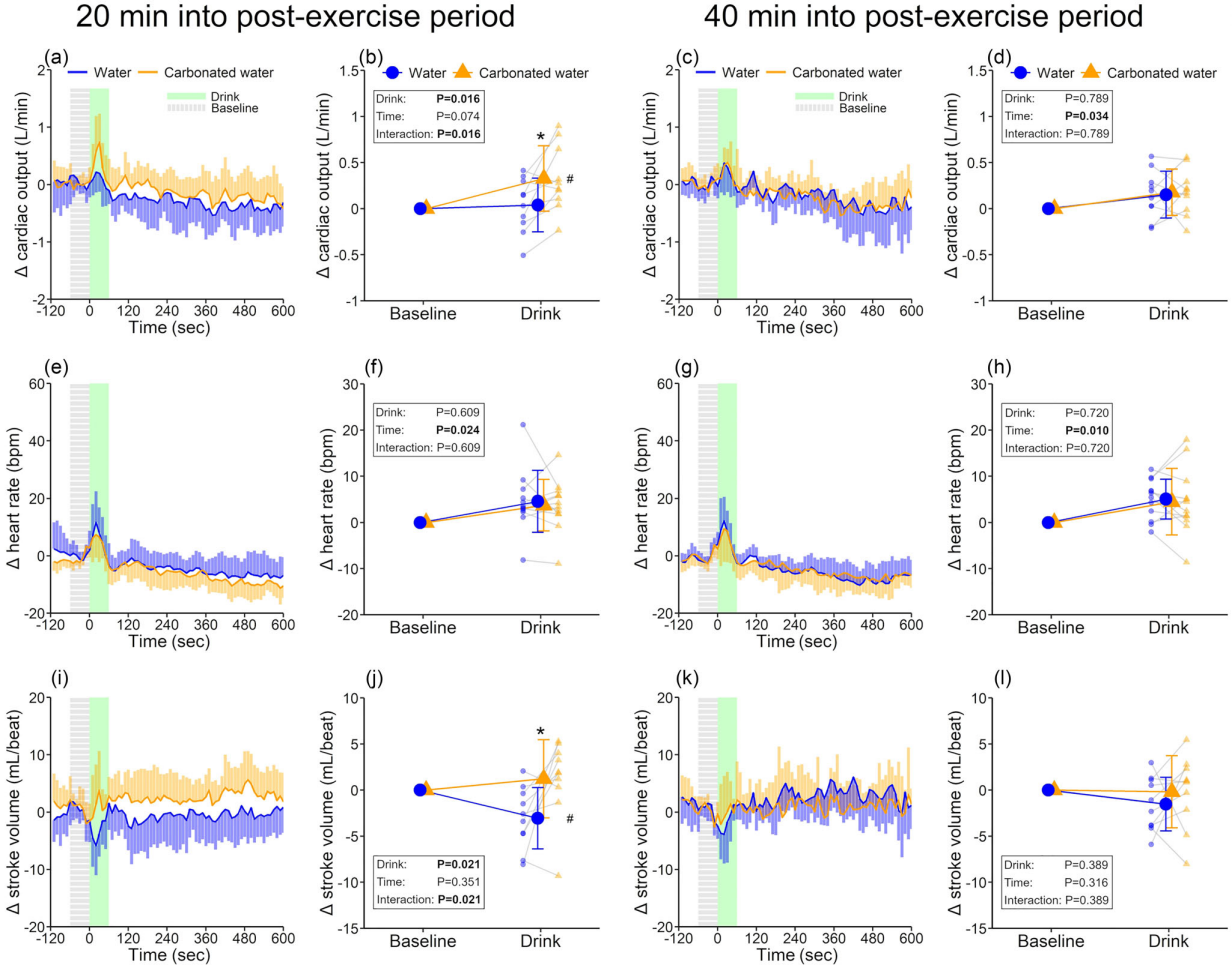
Cardiac responses to drinking. Cardiac output (a‐d, *n* = 10), heart rate (e‐h, *n* = 12) and stroke volume (i‐l, *n* = 10) during drink periods. Every 10‐s data point during drink periods (a, c, e, g, i, k) and the drink‐induced change from baseline (b, d, f, h, j, l) are presented for the three variables. Two‐way repeated measures ANOVA was conducted on the data presented in b, d, f, h, j and l. When an interaction between drink and time was detected, *post hoc* multiple comparisons were carried out with Bonferroni correction. *Versus non‐carbonated water (*P* < 0.05); #versus baseline within a trial (*P* < 0.05). Data are presented as means ± standard deviation with individual data points represented in b, d, f, h, j and l. Bold values indicate statistical significance.

As anticipated, post‐exercise hypotension was observed at the pre‐first drink baseline, but this response vanished at the pre‐second drink baseline (Table 1). Water consumption transiently elevated mean arterial pressure irrespective of the early and late post‐exercise periods (both, *P* ≤ 0.015), with the response being greater by 4.3 ± 2.9 mmHg in the carbonated trial during the early post‐exercise period only (*P* < 0.001, Figure 1a,b). A similar response during the early post‐exercise period was also noted for the middle cerebral artery mean blood velocity (between‐trial difference: 2.2 ± 3.4 cm/s, *P* = 0.046, Figure 1e,f). However, no interaction or main effect of drink and time was detected on the cerebrovascular conductance index (all *P* ≥ 0.328). The observed greater increase in mean arterial pressure in the carbonated water trial is attributed to higher stroke volume (between‐trial difference: 4.3 ± 4.9 mL/beat, *P* = 0.021, Figure 2i,j) and cardiac output (between‐trial difference: 0.29 ± 0.31 L/min, *P* = 0.016, Figure 2a,b), with no differences in total peripheral resistance (Figure S2a). Data collected for 10 min after drinking are presented in Supporting information, Table S3.

### 3.4 Thermoregulatory responses

**FIGURE 3:**
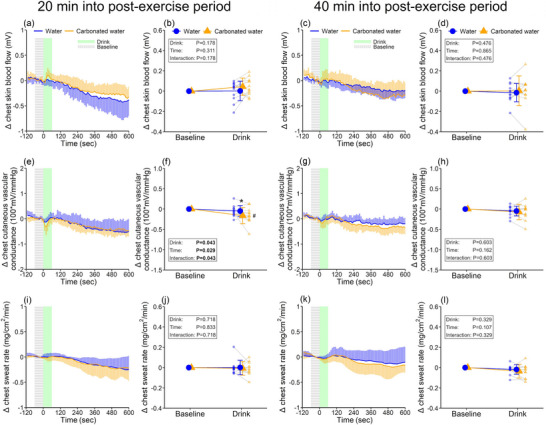
Thermoregulatory responses to drinking. Skin blood flow (a‐d, *n* = 10), cutaneous vascular conductance (e‐h, *n* = 12), and sweating rate (i‐l, *n* = 12) on the chest during drink periods. Every 10‐s data point during drink periods (a, c, e, g, i, k) and the drink‐induced change from baseline (b, d, f, h, j, l) are presented for the three variables. Two‐way repeated measures ANOVA was conducted on the data presented in b, d, f, h, j and l. When an interaction between drink and time was detected, *post hoc* multiple comparisons were carried out with Bonferroni correction. *Versus non‐carbonated water (*P* < 0.05); #versus baseline within a trial (*P* < 0.05). Data are presented as means ± standard deviation with individual data points represented in b, d, f, h, j and l. Bold values indicate statistical significance.

Thermoregulatory and temperature variables were higher at the pre‐first drink baseline compared to the pre‐exercise baseline (Table 2). Subsequently, these elevated values decreased at the pre‐second drink baseline (Table 2). During the early post‐exercise period, chest cutaneous vascular conductance was lower in the carbonated water trial compared to the non‐carbonated one (*P* = 0.043, Figure 3e,f). However, the remaining thermoregulatory and temperature variables did not differ between the two trials throughout the recovery periods (all *P* ≥ 0.101, Figure 3 and Supporting information Figure S2c–l).

### 3.5 Perceptions and mood states

**FIGURE 4:**
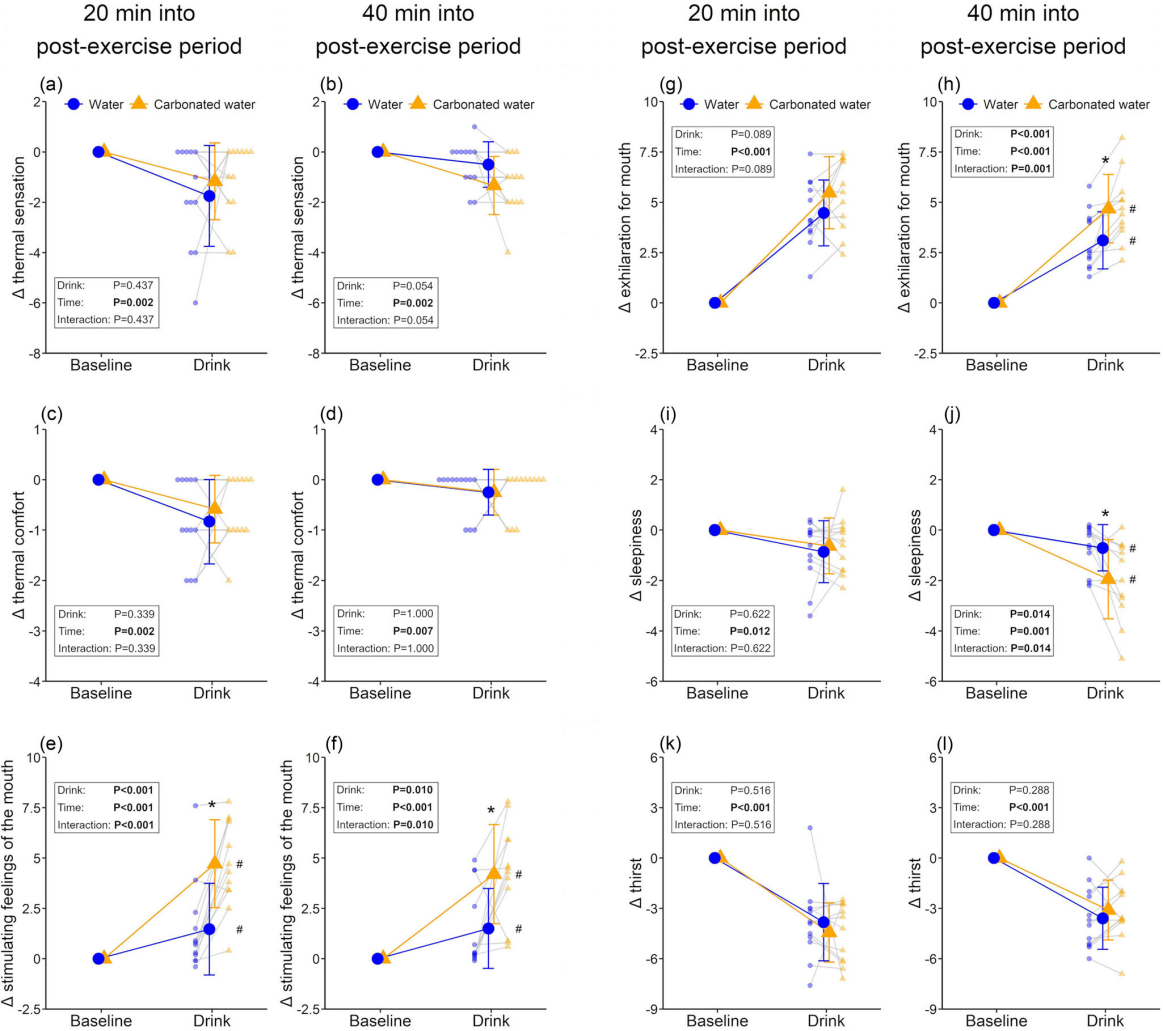
Perceptual and mood state responses to drinking. Thermal sensation (a, b), thermal comfort (c, d), stimulating feeling of the mouth (e, f), exhilaration for mouth (g, h), sleepiness (i, j), and feeling of thirst (k, l) during drink periods (*n* = 12). Drink‐induced changes from baseline are presented for  all variables. Two‐way repeated measures ANOVA was conducted on all data presented. When an interaction between drink and time was detected, *post hoc* multiple comparisons were carried out with Bonferroni correction. *Versus non‐carbonated water (*P* < 0.05); #versus baseline within a trial (*P* < 0.05). Data are presented as means ± standard deviation with individual data points represented.

Thermal comfort, thirst and whole‐body fatigue increased at the pre‐first drink baseline relative to the pre‐exercise baseline, and thermal comfort and whole‐body fatigue decreased from the pre‐first drink to the pre‐second drink baseline (Table 3). Stimulating feeling of the mouth and abdominal fullness in the carbonated water trial increased from the pre‐exercise baseline to the pre‐second drink baseline only (Table 3). Both beverages increased stimulating feelings of the mouth, with this response being greater in the carbonated trial on all occasions (*P* < 0.01) (Figure 4e,f). Drinking increased exhilaration for the mouth while reducing sleepiness regardless of the drink type and period, and these responses were greater with carbonated water during the late post‐exercise period (mouth exhilaration: 3.1 ± 1.4 vs. 4.7 ± 1.7, *P* = 0.001; sleepiness: −0.7 ± 0.91 vs. −1.9 ± 1.6, *P* = 0.014, Figure 4h,j). Drinking reduced thermal sensation, thermal comfort and thirst, while increasing exhilaration for the whole body during the early and late recovery periods (all *P* ≤ 0.007), with these effects being comparable between the two drinks (*P* ≥ 0.054) (Figures 4a–d,k,l and Supporting information Figure S2u, v). Data collected 5 and 10 min after drinking are presented in Supporting information, Table S4.

### 3.6 Hydration status

Exercise reduced plasma volume (Table 4) and body weight (water vs. carbonated water, 2.02 ± 0.78 vs. 1.98 ± 0.89%, *P* = 0.630), while it increased plasma osmolality (Table 4), with the magnitudes being similar between the two drinks. Urine specific gravity (trial: *P* = 0.784, time: *P* = 0.915, interaction: *P* = 0.955) remained comparable between pre‐ and post‐exercise, and it did not differ between the non‐carbonated and carbonated water trials (pre‐exercise: 1.014 ± 0.016 vs. 1.014 ± 0.006, post‐exercise: 1.014 ± 0.006 vs. 1.015 ± 0.006). Estimated total sweat loss was similar between non‐carbonated and carbonated water trials (1.45 ± 0.48 vs. 1.43 ± 0.56 L, *P* = 0.771), with approximately 18% of this loss replenished by consuming either non‐carbonated water or carbonated water.

### 3.7 Respiratory responses

Respiratory variables did not differ between trials during post‐exercise recovery period (Supporting information, Table S1 and Figure S2m–r).

## 4 DISCUSSION

As intended, post‐exercise hypotension was observed following prolonged exercise in the heat, indicating mean arterial pressure reduced by approximately 7 mmHg from the pre‐exercise to post‐exercise state (Table 1). In this scenario, ingestion of both non‐carbonated and carbonated water elevated mean arterial pressure, with carbonated water showing a greater response of approximately a 7 mmHg increase in mean arterial pressure (Figure 1a,b), nearly counteracting the post‐exercise hypotension. A similar pressor response associated with carbonated water ingestion was also identified in our previous study evaluating responses under resting conditions in the heat (37°C) without exercise (Fujii et al., 2022). Considering that the pressor response began immediately upon drinking, we believe it is primarily triggered by the swallowing reflex (Burke et al., 2020), which can raise blood pressure. This response may be amplified by carbonated water, potentially through the activation of TRPV1 channels (Tsuji et al., 2020), as these ion channels are involved in the swallowing reflex (Hossain et al., 2018). Future studies are warranted to delineate whether the aforementioned mechanisms contribute to the pressor response associated with carbonated water ingestion and if other mechanisms, such as acid‐sensing ion channels and carbonic anhydrase, are involved in the pressor response. It should be noted that drink‐induced pressor response was transient, with mean arterial pressure quickly returning to baseline levels (Figure 1a). It may be that the drink‐mediated enhancement of arterial baroreflex control over muscle sympathetic nerve activity (Vianna et al., 2018) remains unaltered even during the post‐exercise period, rapidly diminishing the drink‐induced pressor response.

The greater increase in mean arterial pressure during carbonated water consumption was linked to increased stroke volume (Figure 2i,j) and cardiac output (Figure 2a,b), with no differences in heart rate (Figure 2e,f) and total peripheral resistance (Supporting information, Figure S2a) between trials. Upon closer examination, non‐carbonated water ingestion decreased stroke volume (Figure 2i,j). The precise reason for this response is unclear, but tachycardia associated with drinking (Figure 2e, f) may reduce ventricular filling time and end‐diastolic volume, thus lowering stroke volume. Notably, this response was not observed when consuming carbonated water (Figure 2i,j), leading to a greater increase in cardiac output (Figure 2a,b). Since cutaneous vascular tone, as indicated by chest cutaneous vascular conductance, increased only during the ingestion of carbonated water (Figure 3e,f), if this response occurred over the majority of the lower leg limbs, it might result in reductions in venous pooling, thereby increasing venous return and stroke volume. However, given a similar response was not detected in the forearm cutaneous vascular conductance (Supporting information, Figure S2e), a direct assessment of lower leg cutaneous vascular tone is required in future.

Surprisingly, the pressor response associated with carbonated water ingestion observed during the early recovery period was not detected during the late post‐exercise period (Figure 1c,d), which is in contrast to our original hypothesis. The attenuated pressor response does not seem attributable to habituation from the initial carbonated water ingestion, as we previously found a comparable pressor response between the first and second ingestion of carbonated water (Fujii et al., 2022). The differential pattern of blood pressure response to ingesting carbonated water may be explained by the fact that drinking non‐carbonated water did not decrease stroke volume in contrast to the response observed during the early recovery period (Figure 2k,l). It might be that the influence on stroke volume of tachycardia associated with drinking occurs above a certain level of heart rate, as pre‐drink baseline heart rate was lower during the late versus early recovery period (Table 1). Along these lines, we did not detect a reduction in stroke volume when drinking non‐carbonated water under ambient heat exposure wherein the pre‐drink baseline heart rate was ∼70 bpm (Fujii et al., 2022). Moreover, the heightened chest cutaneous vascular tone observed with carbonated water intake during the early post‐exercise recovery period was not detected during the later stage of the recovery period (Figure 3g,h), underlying the absence of a clear pressor response induced by carbonated water. The lack of a discernible effect of carbonated water ingestion on chest cutaneous vascular conductance could be attributed to the varying levels of cutaneous vascular conductance, which were significantly lower during the late recovery period compared to the early recovery period, thereby reducing the reserve capacity of cutaneous vascular tone to decrease.

The cerebral blood flow index measured in this study reflects changes in mean arterial pressure; both non‐carbonated and carbonated water consumption transiently increased the index regardless of the recovery periods, but the increase was more pronounced with carbonated water during the early recovery period (Figure 1e,f). This aligns with the relationship between cerebral blood flow and mean arterial pressure (Brassard et al., 2021). Moreover, since cardiac output is a potential modulator of cerebral blood flow (Lie et al., 2021; Ogoh et al., 2005), the greater cardiac output induced by carbonated water ingestion may contribute to the increased cerebral blood flow index observed during the early recovery period. Although arterial CO_2_ pressure plays a significant role in regulating cerebral blood flow (Kety & Schmidt, 1948; Ogoh, 2019), carbonated water ingestion is unlikely to have a substantial impact on arterial CO_2_ pressure, as minute ventilation and CO_2_ elimination were similar between the two drink conditions at each baseline and after drink ingestion (Supporting information, Figure S2q,r and Table S1).

Skin blood flow and sweating rate did not increase during water consumption (Figures 3a‐l and Supporting information, S2c‐h) despite the pressor response. In contrast, prior studies reported that applying positive pressure to the lower limbs (Jackson & Kenny, 2003), implementing head‐down tilt (McInnis et al., 2006), or utilizing supine recovery (Kenny et al., 2008) enhanced thermoregulatory responses post‐exercise by elevating mean arterial pressure. Given that the magnitude (≥10 mmHg) and duration (≥60 min) of mean arterial pressure increases induced by the mentioned methods (Jackson & Kenny, 2003; Kenny et al., 2008; McInnis et al., 2006) exceeded those triggered by carbonated water ingestion (i.e., ∼4 mmHg and ∼1 min), the pressor response prompted by carbonated water intake may have been insufficient to influence heat loss responses following exercise in the heat. In line with thermoregulatory responses, core and skin temperatures exhibited similar changes during the post‐exercise recovery period between the two trials (Supporting information, Figure S2i–l).

Carbonated water intake increased mouth exhilaration while reducing sleepiness more than non‐carbonated water intake (Figure 4h,j), suggesting a positive impact on post‐exercise mood. The stimulating feeling of the mouth associated with carbonated water ingestion may have contributed to the heightened mouth exhilaration, as indicated in our prior research (Fujii et al., 2022). Additionally, since acute pain can disrupt sleep (Roehrs & Roth, 2005), the increased stimulating sensation in the mouth might have also mitigated sleepiness. However, despite similar increases in stimulating feeling of the mouth with carbonated water ingestion during both the early and late recovery periods (Figure 4e,f), changes in mood states induced by carbonated water ingestion were only observed during the late stage of the post‐exercise recovery period (Figure 4h,j). Considering that thermal sensation and comfort values were higher (indicating feeling hotter and less comfort) during the early recovery period compared to the late recovery period (Table 3), these factors may have affected the translation of the stimulating feeling of the mouth to mouth exhilaration and sleepiness. Further research is necessary to investigate these possibilities.

### 4.1 Limitations

There are four notable limitations in the present study. First, during water consumption, we could not measure end‐tidal CO_2_ pressure, which is an index of the partial pressure of arterial CO_2_ and can influence cerebral blood flow (Kety & Schmidt, 1948). Consequently, it remains unknown whether the partial pressure of arterial CO_2_ was affected by drinking carbonated water and whether this alteration impacted cerebral blood flow. Nevertheless, the difference in end‐tidal CO_2_ partial pressure between the trials seems to be minimal, as discussed previously. Second, since we did not involve drinking carbonated water before exercise, we cannot directly assess whether the effects of carbonated water were influenced by exercising in a hot environment. Third, we did not employ a no‐drink control trial. Including this trial would have provided more insights, such as whether hydration associated with water or carbonated water modulated responses. Fourth, we did not normalize skin blood flow data to maximal values. Had we assessed this, we might have observed different results.

### 4.2 Practical applications

The current study suggests that carbonated water consumption transiently increases blood pressure and the cerebral blood flow index in the early post‐exercise phase in a hot environment, particularly when post‐exercise hypotension occurs. It should be noted that we tested a single volume of drink only (150 mL for males and 100 mL for females). Future studies are warranted to determine the optimal volume of carbonated water to maximize increases in blood pressure and cerebral blood flow index. Furthermore, continuously consuming carbonated water for an extended duration may be more effective in sustaining increased blood pressure and cerebral blood flow index, which requires direct scrutiny. Although more work is necessary, carbonated water ingestion might be an effective strategy to counter orthostatic syncope and dizziness associated with post‐exercise hypotension.

It is worth noting that the pressor response elicited by carbonated water intake was not observed during the late post‐exercise phase when post‐exercise hypotension was less prominent. Hence carbonated water ingestion does not appear to induce unnecessary pressor responses when blood pressure is within the normal range post‐exercise in the heat. Instead, carbonated water consumption increased mouth exhilaration and reduced sleepiness during the late post‐exercise phase. Consequently, drinking carbonated water may help partially alleviate reductions in work productivity due to occupational heat stress (Flouris et al., 2018) by improving mood state. On the other hand, carbonated water intake does not influence body temperatures, thermal perceptions or hydration markers. Thus, carbonated water ingestion may not exacerbate heat balance, hydration levels, and possibly the risk of heat‐related injuries.

## 5 CONCLUSIONS

We demonstrate that carbonated water intake, an intervention easily accessible to various populations, transiently raises blood pressure, counteracting post‐exercise hypotension, and increases the cerebral blood flow index during the early post‐exercise periods in hot conditions. Furthermore, carbonated water consumption enhances mouth exhilaration and reduces sleepiness during the late stage of post‐exercise in hot ambient temperatures. Additionally, ingesting carbonated water has no adverse impact on thermoregulatory responses, thirst, thermal perceptions or hydration status.

## AUTHOR CONTRIBUTIONS

Conceptualization: Masanobu Kajiki and Naoto Fujii. Data curation: Masanobu Kajiki, Akira Katagiri, Ryoko Matsutake, Yin‐Feng Lai, and Naoto Fujii. Formal analysis: Masanobu Kajiki. Funding acquisition: Hideki Hashimoto, Takeshi Nishiyasu, and Naoto Fujii. Investigation: Masanobu Kajiki, Akira Katagiri, Ryoko Matsutake, Yin‐Feng Lai, and Hideki Hashimoto. Methodology: Masanobu Kajiki and Naoto Fujii. Project administration: Naoto Fujii. Resources: Takeshi Nishiyasu and Naoto Fujii. Supervision: Naoto Fujii. Visualization: Masanobu Kajiki. Writing—original draft: Masanobu Kajiki. Writing—review & editing: Masanobu Kajiki, Akira Katagiri, Ryoko Matsutake, Yin‐Feng Lai, Hideki Hashimoto, Takeshi Nishiyasu, and Naoto Fujii. All authors have read and approved the final version of this manuscript and agree to be accountable for all aspects of the work in ensuring that questions related to the accuracy or integrity of any part of the work are appropriately investigated and resolved. All persons designated as authors qualify for authorship, and all those who qualify for authorship are listed.

## CONFLICT OF INTEREST

The authors declare no competing interest. Asahi Soft Drinks Co., Ltd had no control over the interpretation, writing, or publication of this work. H.H. is an employee of Asahi Soft Drinks Co. Ltd. The results of this study are presented clearly, honestly, and without fabrication, falsification, or inappropriate data manipulation.

## Supporting information

Figures S1 and S2 and Tables S1–S4.

## Data Availability

Data will be made available on request.

## References

[eph13624-bib-0042] Borg, G. (1998). Borg's Perceived Exertion And Pain Scales. Human Kinetics.

[eph13624-bib-0001] Brassard, P. , Labrecque, L. , Smirl, J. D. , Tymko, M. M. , Caldwell, H. G. , Hoiland, R. L. , Lucas, S. J. E. , Denault, A. Y. , Couture, E. J. , & Ainslie, P. N. (2021). Losing the dogmatic view of cerebral autoregulation. Physiological Reports, 9(15), e14982. 34323023 10.14814/phy2.14982 PMC8319534

[eph13624-bib-0002] Burke, P. , Carter, S. G. , Knapman, F. , Patti, J. , Butlin, M. , Gandevia, S. C. , Butler, J. E. , Eckert, D. J. , & Bilston, L. E. (2020). Nocturnal swallowing augments arousal intensity and arousal tachycardia. Proceedings of the National Academy of Sciences, USA, 117(15), 8624–8632. 10.1073/pnas.1907393117 PMC7165485 32229567

[eph13624-bib-0003] Cunha, F. A. , Farinatti, P. , Jones, H. , & Midgley, A. W. (2020). Postexercise hypotension and related hemodynamic responses to cycling under heat stress in untrained men with elevated blood pressure. European Journal of Applied Physiology, 120, 1001–1013. 32189061 10.1007/s00421-020-04340-6 PMC7181414

[eph13624-bib-0004] Des Gachons, C. P. , Avrillier, J. , Gleason, M. , Algarra, L. , Zhang, S. , Mura, E. , Nagai, H. , & Breslin, P. A. (2016). Oral cooling and carbonation increase the perception of drinking and thirst quenching in thirsty adults. PLoS ONE, 11(9), e0162261. 27685093 10.1371/journal.pone.0162261 PMC5042416

[eph13624-bib-0005] Dessirier, J. , Simons, C. T. , Carstens, M. I. , O'Mahony, M. , & Carstens, E. (2000). Psychophysical and neurobiological evidence that the oral sensation elicited by carbonated water is of chemogenic origin. Chemical Senses, 25(3), 277–284. 10866986 10.1093/chemse/25.3.277

[eph13624-bib-0006] Dill, D. B. , & Costill, D. L. (1974). Calculation of percentage changes in volumes of blood, plasma, and red cells in dehydration. Journal of Applied Physiology, 37(2), 247–248. 4850854 10.1152/jappl.1974.37.2.247

[eph13624-bib-0007] Endo, Y. , Torii, R. , Yamazaki, F. , Sagawa, S. , Yamauchi, K. , Tsutsui, Y. , Morikawa, T. , & Shiraki, K. (2001). Water drinking causes a biphasic change in blood composition in humans. Pflugers Archiv: European Journal of Physiology, 442, 362–368. 11484766 10.1007/s004240100555

[eph13624-bib-0008] Endo, Y. , Yamauchi, K. , Tsutsui, Y. , Ishihara, Z. , Yamazaki, F. , Sagawa, S. , & Shiraki, K. (2002). Changes in blood pressure and muscle sympathetic nerve activity during water drinking in humans. The Japanese Journal of Physiology, 52(5), 421–427. 12533246 10.2170/jjphysiol.52.421

[eph13624-bib-0009] Flouris, A. D. , Dinas, P. C. , Ioannou, L. G. , Nybo, L. , Havenith, G. , Kenny, G. P. , & Kjellstrom, T. (2018). Workers' health and productivity under occupational heat strain: A systematic review and meta‐analysis. The Lancet Planetary Health, 2(12), e521–e531. 30526938 10.1016/S2542-5196(18)30237-7

[eph13624-bib-0010] Fujii, N. , Kataoka, Y. , Lai, Y. , Shirai, N. , Hashimoto, H. , & Nishiyasu, T. (2022). Ingestion of carbonated water increases middle cerebral artery blood velocity and improves mood states in resting humans exposed to ambient heat stress. Physiology & Behavior, 255, 113942. 35964802 10.1016/j.physbeh.2022.113942

[eph13624-bib-0011] Fujii, N. , Kenny, G. P. , Amano, T. , Honda, Y. , Kondo, N. , & Nishiyasu, T. (2019). Evidence for TRPV4 channel induced skin vasodilatation through NOS, COX, and KCa channel mechanisms with no effect on sweat rate in humans. European Journal of Pharmacology, 858, 172462. 31216445 10.1016/j.ejphar.2019.172462

[eph13624-bib-0012] Gagnon, D. , Lynn, A. G. , Binder, K. , Boushel, R. C. , & Kenny, G. P. (2012). Mean arterial pressure following prolonged exercise in the heat: Influence of training status and fluid replacement. Scandinavian Journal of Medicine & Science in Sports, 22(5), e99–e107. 22830505 10.1111/j.1600-0838.2012.01506.x

[eph13624-bib-0013] Halliwill, J. R. , Buck, T. M. , Lacewell, A. N. , & Romero, S. A. (2013). Postexercise hypotension and sustained postexercise vasodilatation: What happens after we exercise? Experimental Physiology, 98(1), 7–18. 22872658 10.1113/expphysiol.2011.058065

[eph13624-bib-0014] Halliwill, J. R. , Sieck, D. C. , Romero, S. A. , & Buck, T. M. (2014). Blood pressure regulation X: What happens when the muscle pump is lost? Post‐exercise hypotension and syncope. European Journal of Applied Physiology, 114, 561–578. 24197081 10.1007/s00421-013-2761-1 PMC3944103

[eph13624-bib-0015] Halliwill, J. R. , Taylor, A. J. , & Eckberg, D. L. (1996). Impaired sympathetic vascular regulation in humans after acute dynamic exercise. The Journal of Physiology, 495(1), 279–288. 8866370 10.1113/jphysiol.1996.sp021592 PMC1160743

[eph13624-bib-0016] Hardy, J. D. , Du Bois, E. F. , & Soderstrom, G. F. (1938). The technic of measuring radiation and convection: One figure. The Journal of Nutrition, 15(5), 461–475.

[eph13624-bib-0017] Horiuchi, M. , & Oliver, S. J. (2023). Greater post‐exercise hypotension in healthy young untrained men after exercising in a hot compared to a temperate environment. Journal of Thermal Biology, 117, 103683. 37625342 10.1016/j.jtherbio.2023.103683

[eph13624-bib-0018] Hossain, M. Z. , Ando, H. , Unno, S. , Masuda, Y. , & Kitagawa, J. (2018). Activation of TRPV1 and TRPM8 channels in the larynx and associated laryngopharyngeal regions facilitates the swallowing reflex. International Journal of Molecular Sciences, 19(12), 4113. 30567389 10.3390/ijms19124113 PMC6321618

[eph13624-bib-0019] Jackson, D. N. , & Kenny, G. P. (2003). Upright lower body positive pressure application attenuates elevated post‐exercise resting thresholds for cutaneous vasodilation and sweating in humans. Journal of Applied Physiology, 95(1), 121–128. 12611766 10.1152/japplphysiol.01006.2002

[eph13624-bib-0020] Janal, M. N. , Colt, E. W. , Clark, W. C. , & Glusman, M. (1984). Pain sensitivity, mood and plasma endocrine levels in man following long‐distance running: Effects of naloxone. Pain, 19(1), 13–25. 6330643 10.1016/0304-3959(84)90061-7

[eph13624-bib-0021] Kenefick, R. W. , & Cheuvront, S. N. (2012). Hydration for recreational sport and physical activity. Nutrition Reviews, 70(suppl_2), S137–S142. 23121349 10.1111/j.1753-4887.2012.00523.x

[eph13624-bib-0022] Kenny, G. P. , Gagnon, D. , Jay, O. , McInnis, N. H. , Journeay, W. S. , & Reardon, F. D. (2008). Can supine recovery mitigate the exercise intensity dependent attenuation of post‐exercise heat loss responses? Applied Physiology, Nutrition, and Metabolism, 33(4), 682–689. 10.1139/H08-053 18641710

[eph13624-bib-0023] Kenny, G. P. , & Journeay, W. S. (2010). Human thermoregulation: Separating thermal and nonthermal effects on heat loss. Frontiers in Bioscience‐Landmark, 15(1), 259–290. 10.2741/3620 20036820

[eph13624-bib-0024] Kenny, G. P. , & McGinn, R. (2017). Restoration of thermoregulation after exercise. Journal of Applied Physiology, 122(4), 933–944. 27881668 10.1152/japplphysiol.00517.2016

[eph13624-bib-0025] Kety, S. S. , & Schmidt, C. F. (1948). The effects of altered arterial tensions of carbon dioxide and oxygen on cerebral blood flow and cerebral oxygen consumption of normal young men. The Journal of Clinical Investigation, 27(4), 484–492. 16695569 10.1172/JCI101995 PMC439519

[eph13624-bib-0026] Kubota, S. , Endo, Y. , Kubota, M. , Miyazaki, H. , & Shigemasa, T. (2022). The pressor response to the drinking of cold water and cold carbonated water in healthy younger and older adults. Frontiers in Neurology, 12, 2417. 10.3389/fneur.2021.788954 PMC8793880 35095733

[eph13624-bib-0027] Lie, S. L. , Hisdal, J. , & Høiseth, L. Ø. (2021). Cerebral blood flow velocity during simultaneous changes in mean arterial pressure and cardiac output in healthy volunteers. European Journal of Applied Physiology, 121, 2207–2217. 33890157 10.1007/s00421-021-04693-6 PMC8260418

[eph13624-bib-0028] McInnis, N. H. , Journeay, W. S. , Jay, O. , Leclair, E. , & Kenny, G. P. (2006). 15° Head‐down tilt attenuates the postexercise reduction in cutaneous vascular conductance and sweating and decreases esophageal temperature recovery time. Journal of Applied Physiology, 101(3), 840–847. 16741261 10.1152/japplphysiol.00382.2006

[eph13624-bib-0029] Ogoh, S. (2019). Interaction between the respiratory system and cerebral blood flow regulation. Journal of Applied Physiology, 127(5), 1197–1205. 30920887 10.1152/japplphysiol.00057.2019

[eph13624-bib-0030] Ogoh, S. , Brothers, R. M. , Barnes, Q. , Eubank, W. L. , Hawkins, M. N. , Purkayastha, S. , O‐Yurvati, A. , & Raven, P. B. (2005). The effect of changes in cardiac output on middle cerebral artery mean blood velocity at rest and during exercise. The Journal of Physiology, 569(2), 697–704. 16210355 10.1113/jphysiol.2005.095836 PMC1464249

[eph13624-bib-0031] Ramanathan, N. L. (1964). A new weighting system for mean surface temperature of the human body. Journal of Applied Physiology, 19(3), 531–533. 14173555 10.1152/jappl.1964.19.3.531

[eph13624-bib-0032] Roehrs, T. , & Roth, T. (2005). Sleep and pain: Interaction of two vital functions. Seminars in Neurology, 25(1), 106–116. 15798943 10.1055/s-2005-867079

[eph13624-bib-0033] Schlader, Z. J. , Gagnon, D. , Adams, A. , Rivas, E. , Cullum, C. M. , & Crandall, C. G. (2015). Cognitive and perceptual responses during passive heat stress in younger and older adults. American Journal of Physiology‐Regulatory, Integrative and Comparative Physiology, 308(10), R847–R854. 25786484 10.1152/ajpregu.00010.2015 PMC4436980

[eph13624-bib-0034] Smit, H. J. , Cotton, J. R. , Hughes, S. C. , & Rogers, P. J. (2004). Mood and cognitive performance effects of “energy” drink constituents: Caffeine, glucose and carbonation. Nutritional Neuroscience, 7(3), 127–139. 15526987 10.1080/10284150400003041

[eph13624-bib-0035] Tsuji, K. , Tsujimura, T. , Sakai, S. , Suzuki, T. , Yoshihara, M. , Nagoya, K. , Magara, J. , Satoh, Y. , & Inoue, M. (2020). Involvement of capsaicin‐sensitive nerves in the initiation of swallowing evoked by carbonated water in anesthetized rats. American Journal of Physiology‐Gastrointestinal and Liver Physiology, 319(5), G564–G572. 32878469 10.1152/ajpgi.00233.2020

[eph13624-bib-0036] Vianna, L. C. , Fernandes, I. A. , Martinez, D. G. , Teixeira, A. L. , Silva, B. M. , Fadel, P. J. , & Nóbrega, A. C. (2018). Water drinking enhances the gain of arterial baroreflex control of muscle sympathetic nerve activity in healthy young humans. Experimental Physiology, 103(10), 1318–1325. 30055008 10.1113/EP087095

[eph13624-bib-0037] Wakisaka, S. , Nagai, H. , Mura, E. , Matsumoto, T. , Moritani, T. , & Nagai, N. (2012). The effects of carbonated water upon gastric and cardiac activities and fullness in healthy young women. Journal of Nutritional Science and Vitaminology, 58(5), 333–338. 23327968 10.3177/jnsv.58.333

[eph13624-bib-0038] Wesseling, K. H. , Jansen, J. R. , Settels, J. J. , & Schreuder, J. J. (1993). Computation of aortic flow from pressure in humans using a nonlinear, three‐element model. Journal of Applied Physiology, 74(5), 2566–2573. 8335593 10.1152/jappl.1993.74.5.2566

[eph13624-bib-0039] West, A. M. , Tarrier, J. , Hodder, S. , & Havenith, G. (2019). Sweat distribution and perceived wetness across the human foot: The effect of shoes and exercise intensity. Ergonomics, 62(11), 1450–1461. 31422758 10.1080/00140139.2019.1657185

[eph13624-bib-0040] Willmott, A. G. , Hayes, M. , James, C. A. , Gibson, O. R. , & Maxwell, N. S. (2020). Heat acclimation attenuates the increased sensations of fatigue reported during acute exercise‐heat stress. Temperature, 7(2), 178–190. 10.1080/23328940.2019.1664370 PMC7518764 33015245

