# Supplementary material for: Ingesting carbonated water post‐exercise in the heat transiently ameliorates hypotension and enhances mood state
Source: Exp Physiol. 2024 Aug 14;109(10):1683–97. doi: 10.1113/EP091925 (PMC11442846; doi:10.1113/EP091925)
Supplement: Supplementary file 1 — Figures S1 and S2 and Tables S1–S4. [file EPH-109-1683-s001.docx]

**Figure caption**

**Figure S1**. A schematic overview of the experimental protocol.

**Figure S2**. Drink induced changes in total peripheral resistance (a, b), forearm skin blood flow (c, d), forearm cutaneous vascular conductance (e, f), forearm sweat rate (g, h), rectal temperature (i, j), mean skin temperature (k, l), minute ventilation (m, n), O_2_ uptake (o, p), CO_2_ elimination (q, r), abdominal fullness (s, t), exhilaration for the whole-body (u, v) and whole-body fatigue (w, x) in water and carbonated water are presented (n=12). Baseline, data averaged over 1-min immediately before drinking; Drink, 1-min average data during drinking. Two-way repeated measure ANOVA was conducted on all data presented. Data are presented as mean ± standard deviation with individual data points represented. Bold values indicate statistical significance.

**Figure S1**


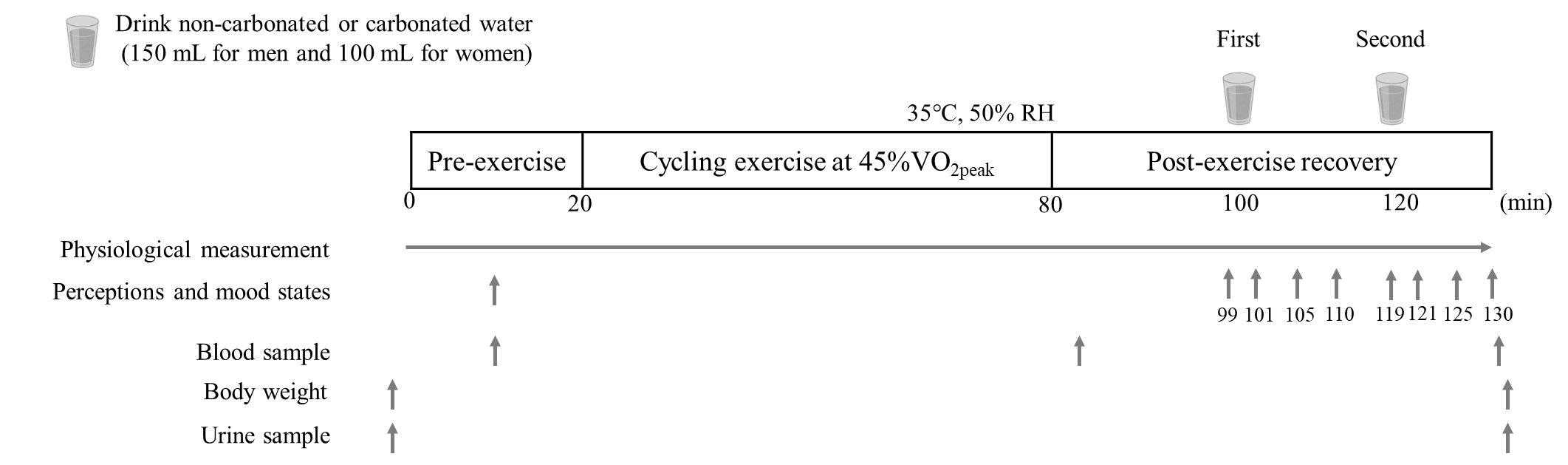


**Figure S2**


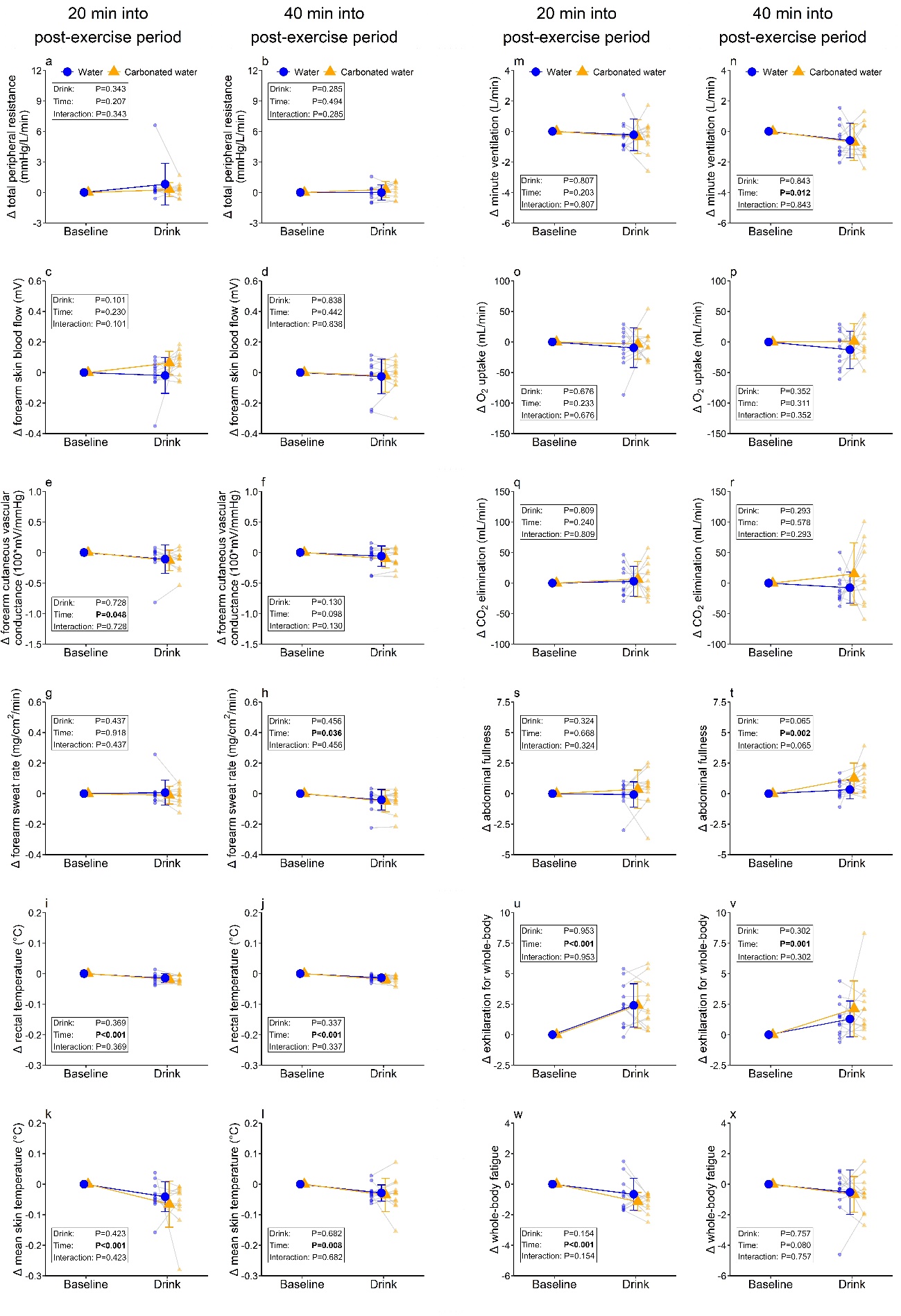


Table S1. Baseline respiratory variables measured immediately before exercise and ingesting first and second drinks

|  |  | t-test P value |  |  | ANOVA P value | | |
| --- | --- | --- | --- | --- | --- | --- | --- |
|  | Pre-exercise |  | Pre-first drink | Pre-second drink | Trial | Time | Interaction |
| Minute ventilation (L/min) |  | 0.061 |  |  | 0.200 | 0.710 | 0.099 |
| Water | 11.4 ± 1.2 |  | 10.2 ± 2.5 | 9.5 ± 2.4 |  | | |
| Carbonated water | 12.2 ± 2.0 |  | 10.0 ± 2.4 | 10.4 ± 1.6 |  |  |  |
| O_2_ uptake (mL/min) |  | 0.739 |  |  | 0.582 | **0.017** | 0.569 |
| Water | 274 ± 36 |  | 280 ± 52 | 263 ± 54 |  | | |
| Carbonated water | 278 ± 48 |  | 272 ± 44 | 261 ± 35 |  |  |  |
| CO_2_ elimination (mL/min) |  | 0.131 |  |  | 0.223 | 0.226 | 0.502 |
| Water | 238 ± 29 |  | 234 ± 40 | 220 ± 47 |  | | |
| Carbonated water | 256 ± 47 |  | 239 ± 44 | 236 ± 33 |  |  |  |
| Respiratory exchange ratio |  | **0.003** |  |  | 0.055 | 0.555 | 0.523 |
| Water | 0.86 ± 0.03 |  | 0.84 ± 0.06 | 0.83 ± 0.08 |  | | |
| Carbonated water | 0.92 ± 0.06 |  | 0.88 ± 0.09 | 0.83 ± 0.06 |  |  |  |
| End-tidal CO_2_ partial pressure (mmHg) |  | 0.679 |  |  | 0.827 | 0.248 | 0.690 |
| Water | 36.3 ± 2.1 |  | 37.3 ± 2.7 | 38.1 ± 1.8 |  | | |
| Carbonated water | 36.0 ± 18 |  | 37.3 ± 1.6 | 37.8 ± 2.8 |  |  |  |
| Values are presented as means ±SD; n = 11. Values at Pre-exercise, Pre-first drink and Pre-second drink were obtained by averaging data over the last 5-min of pre-exercise resting period and 19-20 min and 39-40 min into post-exercise periods. Data of pre-exercise were analyzed by a two-tailed paired t-test, whereas data obtained post-exercise were analyzed by two-way repeated measure ANOVA. These separate analyses were necessary as respiratory data were collected via facemask at Pre-exercise, whereas those during post-exercise were obtained via mouth piece. Bold values indicate statistical significance. | | | | | | | |

Table S2. End-exercise variables

| End exercise | | | |
| --- | --- | --- | --- |
|  | Water | Carbonated water | t-test P value |
| Mean arterial pressure (mmHg) | 96.8 ± 28.8 | 95.4 ± 22.1 | 0.722 |
| Middle cerebral artery mean blood velocity (cm/s) | 57.3 ± 14.3 | 55.7 ± 14.2 | 0.256 |
| Cardiac output (L/min) | 12.6 ± 3.1 | 12.8 ± 3.2 | 0.711 |
| Heart rate (bpm) | 146.9 ± 18.4 | 145.9 ± 18.6 | 0.742 |
| Stroke volume (mL/beat) | 85.3 ± 18.1 | 86.5 ± 17.0 | 0.760 |
| Chest skin blood flow (mV) | 2.76 ± 0.66 | 2.84 ± 0.97 | 0.765 |
| Chest cutaneous vascular conductance (100*mV/mmHg) | 2.99 ± 0.89 | 2.98 ± 0.84 | 0.982 |
| Chest sweat rate (mg/cm^2^/min) | 1.71 ± 0.94 | 1.71 ± 0.46 | 0.967 |
| Forearm skin blood flow (mV) | 2.23 ± 0.54 | 2.59 ± 0.84 | **0.036** |
| Forearm cutaneous vascular conductance (100*mV/mmHg) | 2.42 ± 0.77 | 2.77 ± 0.98 | 0.062 |
| Forearm sweat rate (mg/cm^2^/min) | 1.46 ± 0.59 | 1.50 ± 0.63 | 0.690 |
| Rectal temperature (℃) | 38.41 ± 0.46 | 38.38 ± 0.52 | 0.762 |
| Mean skin temperature (℃) | 37.19 ± 0.45 | 36.96 ± 0.45 | 0.111 |
| Minute ventilation (L/min) | 42.7 ± 11.4 | 43.6 ± 11.0 | 0.271 |
| O_2_ uptake (mL/min) | 1284 ± 340 | 1313 ± 314 | 0.061 |
| CO_2_ elimination (mL/min) | 1148 ± 328 | 1198 ± 322 | **0.007** |
| Respiratory exchange ratio | 0.89 ± 0.03 | 0.91 ± 0.04 | 0.111 |
| End-tidal CO_2_ partial pressure (mmHg) | 40.7 ± 2.3 | 40.7 ± 2.0 | 0.951 |
| Rating of perceived exertion | 14.8 ± 2.0 | 15.2 ± 2.1 | 0.470 |
| Thermal sensation | 11.9 ± 4.6 | 12.7 ± 5.6 | 0.275 |
| Thermal comfort | 5.2 ± 1.6 | 5.1 ± 1.7 | 0.754 |
| End exercise: the last 5 min during exercise. Data were analyzed by a two-tailed paired t-test. Bold values indicate statistical significance. | | | |

Table S3. Drink mediated changes in cardiovascular variables measured at 5 min and 10 min into post-drink periods

|  | Post first drink | | Post second drink | |
| --- | --- | --- | --- | --- |
|  | 5 min | 10 min | 5 min | 10 min |
| Mean arterial pressure (mmHg) |  |  |  |  |
| Water | 0.7 ± 2.5 | 1.8 ± 2.6 | -1.6 ± 2.8 | -2.8 ± 6.9 |
| Carbonated water | 0.6 ± 2.7 | 0.4 ± 3.7 | -0.1 ± 2.1 | -0.3 ± 2.6 |
| Middle cerebral artery mean blood velocity (cm/s) |  |  |  |  |
| Water | 0.7 ± 4.0 | 1.7 ± 4.9 | 0.6 ± 3.6 | -0.2 ± 4.0 |
| Carbonated water | 0.6 ± 3.4 | 1.0 ± 3.4 | 0.8 ± 1.7 | 1.0 ± 3.3 |
| Cardiac output (L/min) (n=10) |  |  |  |  |
| Water | -0.30 ± 0.5 | -0.39 ± 0.35 | -0.17 ± 0.21 | -0.36 ± 0.51 |
| Carbonated water | -0.17 ± 0.3 | -0.27 ± 0.38 | -0.16 ± 0.23 | -0.29 ± 0.38 |
| Heart rate (bpm) |  |  |  |  |
| Water | -6.4 ± 3.1 | -9.0 ± 4.1 | -5.7 ± 4.0 | -7.0 ± 5.3 |
| Carbonated water | -5.8 ± 5.4 | -10.0 ± 4.4 | -3.1 ± 5.7 | -5.4 ± 5.4 |
| Stroke volume (mL/beat) (n=10) |  |  |  |  |
| Water | -0.6 ± 5.5 | 0.3 ± 3.8 | 2.4 ± 2.6 | 1.5 ± 4.6 |
| Carbonated water | 1.9 ± 4.1 | 2.8 ± 4.3 | 1.4 ± 4.4 | 1.1 ± 4.3 |
| Total peripheral resistance (mmHg/L/min) (n=10) |  |  |  |  |
| Water | 0.72 ± 1.06 | 1.01 ± 0.73 | 0.03 ± 1.13 | 0.19 ± 1.56 |
| Carbonated water | 0.51 ± 1.00 | 0.73 ± 0.91 | 0.39 ± 0.55 | 0.69 ± 0.85 |
| Values are presented as means ±SD; n = 12 unless otherwise indicated. Values were obtained by averaging data from 4-5 min (5 min) and 9-10 min (10 min) after first and second drinks, respectively. | | | | |

Table S4. Drink mediated changes in perceptions and mood states measured at 5 min and 10 min into post-drink periods

|  |  |  | Post-drink period | | ANOVA P value | | |
| --- | --- | --- | --- | --- | --- | --- | --- |
|  |  | Pre-drink baseline | 5 min | 10 min | Trial | Time | Interaction |
| Thermal sensation | |  |  |  |  |  |  |
| First drink *a,b* | Water | 0 ± 0 | -2.3 ± 2.3 | -2.2 ± 2.0 | 0.244 | **<0.001** | 0.074 |
|  | Carbonated water | 0 ± 0 | -0.9 ± 1.2 | -1.9 ± 1.8 |  |  |  |
| Second drink *a* | Water | 0 ± 0 | -0.6 ± 1.0 | -0.8 ± 1.4 | **0.039** | **0.005** | 0.127 |
|  | Carbonated water | 0 ± 0 | -1.4 ± 1.5 | -1.3 ± 1.4 |  |  |  |
| Thermal comfort |  |  |  |  |  |  |  |
| First drink *a,b* | Water | 0 ± 0 | -0.6 ± 0.8 | -0.9 ± 1.0 | 0.852 | **0.001** | 0.518 |
|  | Carbonated water | 0 ± 0 | -0.6 ± 0.7 | -0.7 ± 0.6 |  |  |  |
| Second drink *a,b* | Water | 0 ± 0 | -0.4 ± 0.5 | -0.3 ± 0.5 | 0.838 | **0.001** | 0.478 |
|  | Carbonated water | 0 ± 0 | -0.3 ± 0.5 | -0.4 ± 0.5 |  |  |  |
| Thirst |  |  |  |  |  |  |  |
| First drink *a,b* | Water | 0 ± 0 | -1.9 ± 2.7 | -1.3 ± 2.3 | 0.245 | **<0.001** | 0.278 |
|  | Carbonated water | 0 ± 0 | -3.2 ± 1.7 | -2.3 ± 1.7 |  |  |  |
| Second drink *a,b* | Water | 0 ± 0 | -1.6 ± 1.7 | 0.9 ± 1.3 | 0.298 | **<0.001** | 0.564 |
|  | Carbonated water | 0 ± 0 | 2.0 ± 1.6 | 1.4 ± 1.1 |  |  |  |
| Stimulating feelings of the mouth | |  |  |  |  |  |  |
| First drink *c* | Water | 0 ± 0 | 0.7 ± 1.4 | 0.2 ± 0.6 | 0.138 | **0.026** | 0.310 |
|  | Carbonated water | 0 ± 0 | 1.3 ± 1.5 | 0.7 ± 1.3 |  |  |  |
| Second drink *a,b* | Water | 0 ± 0 | 0.7± 1.3 | 0.4 ± 0.6 | 0.327 | **<0.001** | 0.573 |
|  | Carbonated water | 0 ± 0 | 1.1 ± 1.0 | 0.8 ± 1.0 |  |  |  |
| Abdominal fullness |  |  |  |  |  |  |  |
| First drink | Water | 0 ± 0 | -0.3 ± 1.3 | -0.6 ± 1.2 | **0.011** | 0.625 | **0.009** |
|  | Carbonated water | 0 ± 0 | 0.8 ± 1.2 * | 0.8 ± 1.8 * |  |  |  |
| Second drink *b* | Water | 0 ± 0 | 0.6 ± 1.2 | 0.2 ± 0.9 | 0.614 | **0.006** | 0.401 |
|  | Carbonated water | 0 ± 0 | 0.6 ± 0.9 | 0.7 ± 1.0 |  |  |  |
| Exhilaration for the mouth |  |  |  |  |  |  |  |
| First drinking *a,b,c* | Water | 0 ± 0 | 2.5 ± 1.5 | 1.4 ± 1.1 | 0.254 | **<0.001** | 0.138 |
|  | Carbonated water | 0 ± 0 | 2.5 ± 1.6 | 2.1 ± 1.8 |  |  |  |
| Second drink | Water | 0 ± 0 | 1.1 ± 1.8 | 0.5 ± 1.1 | 0.057 | **0.016** | 0.355 |
|  | Carbonated water | 0 ± 0 | 1.6 ± 1.8 | 1.1 ± 1.6 |  |  |  |
| Exhilaration for the whole-body | |  |  |  |  |  |  |
| First drink *a* | Water | 0 ± 0 | 1.1 ± 1.3 | 0.7 ± 1.4 | 0.271 | **0.002** | 0.313 |
|  | Carbonated water | 0 ± 0 | 1.6 ± 1.4 | 0.9 ± 1.4 |  |  |  |
| Second drink *a,b* | Water | 0 ± 0 | 1.0 ± 1.2 | 0.4 ± 0.8 | 0.545 | **0.007** | 0.617 |
|  | Carbonated water | 0 ± 0 | 1.4 ± 1.9 | 0.6 ± 1.1 |  |  |  |
| Whole-body fatigue | |  |  |  |  |  |  |
| First drink *a,b* | Water | 0 ± 0 | -0.1 ± 1.0 | -0.7 ± 1.4 | 0.159 | **<0.001** | 0.09 |
|  | Carbonated water | 0 ± 0 | -1.1 ± 0.8 | -1.0 ± 0.7 |  |  |  |
| Second drink | Water | 0 ± 0 | -0.02 ± 0.9 | 0.1 ± 1.0 | 0.438 | 0.527 | 0.646 |
|  | Carbonated water | 0 ± 0 | -0.3 ± 0.8 | -0.2 ± 0.9 |  |  |  |
| Sleepiness | |  |  |  |  |  |  |
| First drink | Water | 0 ± 0 | -0.3 ± 1.0 | 0.2 ± 1.9 | 0.828 | 0.468 | 0.967 |
|  | Carbonated water | 0 ± 0 | -0.2 ± 1.8 | 0.3 ± 1.3 |  |  |  |
| Second drink *a,c* | Water | 0 ± 0 | -0.3 ± 1.2 | 0.8 ± 1.3 | **0.032** | **<0.001** | 0.110 |
|  | Carbonated water | 0 ± 0 | -1.2 ± 1.0 | -0.2 ± 0.6 |  |  |  |
| Values are presented as means ±SD; n = 12. Data were analyzed by two-way repeated measure ANOVA. When an interaction between trial and time was detected, post hoc multiple comparisons were performed with Bonferroni correction. *: vs. non-carbonated water at same time point (P < 0.05), *a*: Baseline vs 5 min (P < 0.05), *b*: Baseline vs 10 min (P < 0.05), *c*: 5 min vs 10 min (P < 0.05). Bold values indicate statistical significance. | | | | | | | |
